# The Eyeball Test

**DOI:** 10.31486/toj.23.5035

**Published:** 2023

**Authors:** Bobby D. Nossaman

**Affiliations:** ^1^ Department of Anesthesiology and Perioperative Medicine, Ochsner Clinic Foundation, New Orleans, LA; ^2^ The University of Queensland Medical School, Ochsner Clinical School, New Orleans, LA

## THE RECOMMENDATION FOR INTRAVENOUS FLUIDS DURING SURGERY BEGINS IN NEW ORLEANS

The administration of intravenous fluids, crystalloids and colloids, has been an integral part of care for most surgical procedures, yet the replacement volume needed to maintain intravascular normovolemia continues to be a daily issue for most anesthesiologists.^1^ Although surgical procedures advanced following the introduction of ether anesthesia in the 1860s,^2^ intravenous fluids were only first proposed for surgery in 1924 by Dr Rudolph Matas of New Orleans to “restore the vascular equilibrium and rally the patient until the defensive cardiovascular mechanism has had time to assert itself*….*”^3^ In Matas’ day, the common methods for rehydrating patients unable to tolerate oral fluids were proctoclysis or hypodermoclysis, methods that were not always successful. Matas recommended the venoclysis route, with the intravenous infusion continued *guttatim* for as long as circulatory doubt existed.^3^ However, case descriptions of intravenous hydration included patients developing congestion of the lungs and heart when fluid was rapidly administered, hence Matas’ recommendation of a “drop by drop” rate of infusion.^3-5^ These early descriptions were observed in critically ill patients, and the understanding of ischemia-reperfusion syndrome may not have been known.^3-5^

## THE DEBATE CONTINUES: WET OR DRY?

What is the right fluid balance for pulmonary resection procedures or for surgical procedures in general? The study of perioperative fluid therapy has increased in recent years as studies show that a fluid administration strategy can influence postoperative outcomes.^6-12^ Historically, the development of postpneumonectomy pulmonary edema as originally described by Zeldin, Normandin, and colleagues^13^ demonstrated higher complication rates when associated with excessive intravenous fluid therapy. Consequently, Zeldin et al and others recommended restricting the administration of intravenous fluids during pulmonary surgery.^13-17^ Other researchers, however, could not associate postpneumonectomy pulmonary edema with excessive intravenous fluid administration.^18^ In contrast, restricting fluid therapy risks inadequate organ perfusion with worse postoperative outcomes.^19^ Although the goal is to maintain intravascular normovolemia,^20,21^ the classic measures of intraoperative systemic hemodynamics and urine output do not provide satisfactory measures of intravascular normovolemia.^22^ Furthermore, investigations with the use of complex monitoring instruments to measure perioperative intravascular volume status have not answered this need,^22^ hence the continued use of guidelines rather than individualization measures to maintain intravascular normovolemia during the surgical procedure.^15-17^

## THE USE OF VOLUMETRIC CURVES TO STUDY OUTCOMES

Bellamy^20^ and Bundgaard-Nielsen, Secher, and Kehlet^21^ proposed using volumetric dose-response curves to associate perioperative fluid administration with the incidence of postoperative morbidities. We agree with that concept. The authors proposed that the volumetric curves would be shaped in the form of a U, with the limbs of the U representing increased morbidities and the optimum fluid administration range located at the bottom of the U.^20,21^ The approach in our study was to examine the dose-response relationships to previously reported adverse events to determine the optimum intraoperative fluid administration rates^23^ and then compare these findings to other lung resection studies that reported their analyses with conventional summation statistics.^24-27^

**Figure 1.**
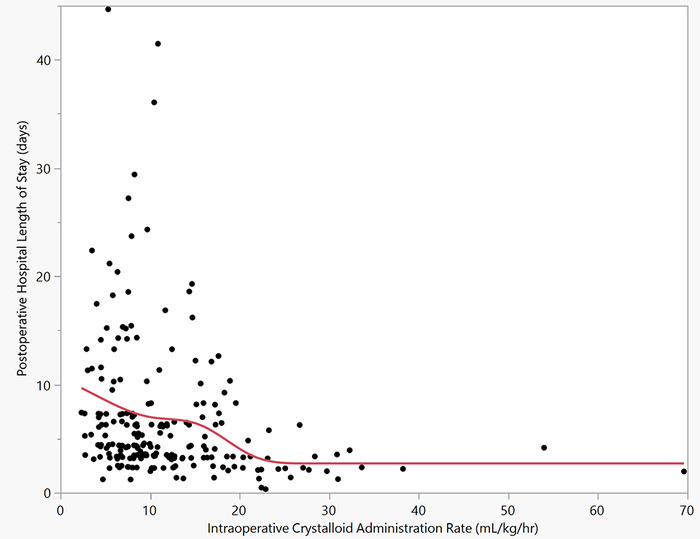
Knotted spline graph showing the duration of postoperative hospital length of stay relative to the intraoperative crystalloid administration rate (mL/kg/hr).

In our study, the associations of the intraoperative crystalloid administration rate (mL/kg/hr) to previously reported adverse events were in the shapes of J-curves (see Figures 1 through 7 in our paper “Rate of Intraoperative Crystalloid Administration During Thoracic Surgery Is Causal in Reducing Postoperative Hospital Length of Stay,” doi.org/10.31486/toj.22.0113)^23^ rather than the hypothesized U-curves.^20,21^ J-curves are not a new concept, as they have been used in the medical literature to optimize blood pressure therapy^28-31^; to examine relationships between alcohol intake and noncardiogenic strokes,^32,33^ between plasma low-density lipoprotein concentrations and recurrent coronary events,^34^ and between pediatric serum lead levels and intellectual development^35-37^; and to investigate the role of body mass index on the incidences of cancer, hip fractures, and mortality.^38-41^

## CAN THE SHAPE OF CURVES ALLOW PREDICTION?

In our study, postoperative hospital length of stay was fitted to intraoperative crystalloid administration rate (Figure 1 in our paper “Rate of Intraoperative Crystalloid Administration During Thoracic Surgery Is Causal in Reducing Postoperative Hospital Length of Stay,” doi.org/10.31486/toj.22.0113).^23^ However, when using a different statistical fitting tool, knotted splines on this same association, a different fit is obtained (Figure 1 in this editorial). This fitted line is now under less summation constraints, and 3 patterns begin to emerge. In the intraoperative crystalloid administration rate range 0 to 10 mL/kg/hr, a higher mean of postoperative hospital length of stay is observed and the highest number of outliers (defined below). A second group between 10 to 20 mL/kg/hr has a lower fitted mean and a lower number of outliers with fewer extreme values when compared to the 0 to 10 mL/kg/hr group. A third group develops once the fitted line crosses into the intraoperative crystalloid administration of 20 mL/kg/hr. The associations viewed with knotted splines analysis suggest ideal administration rates of 20 to 30 mL/kg/hr, as no further reduction in postoperative hospital length of stay was observed: an eyeball test.^42^ However, these observations need further investigation.

**Figure 2.**
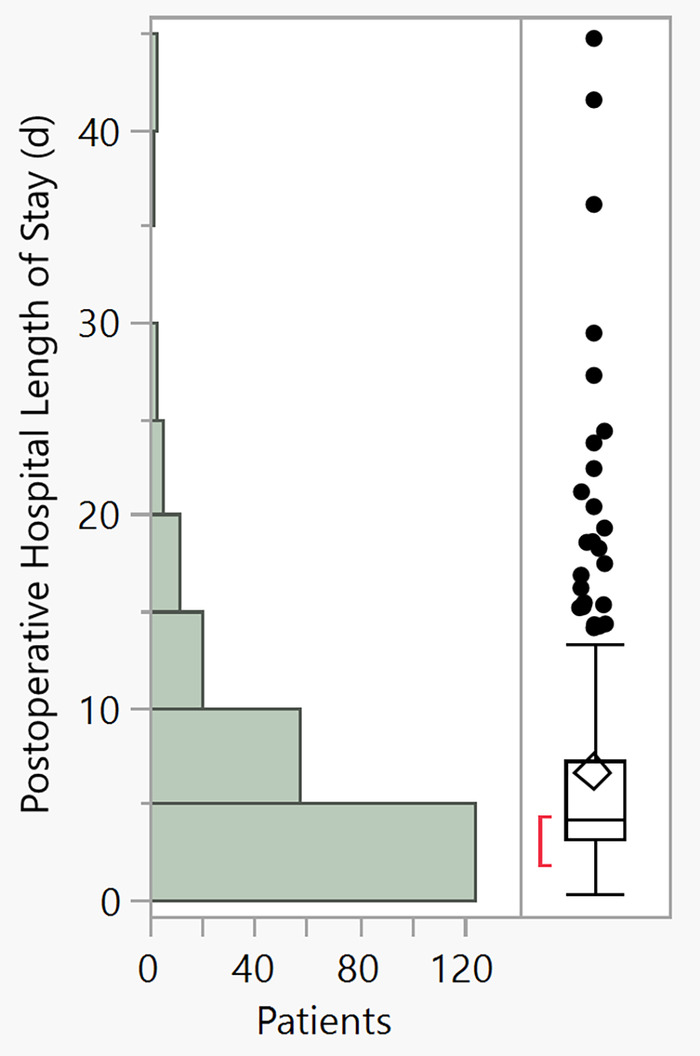
**Histogram with outlier box plot of postoperative hospital length of stay in 222 consecutive patients following thoracic surgery.** d, days.

**Figure 3.**
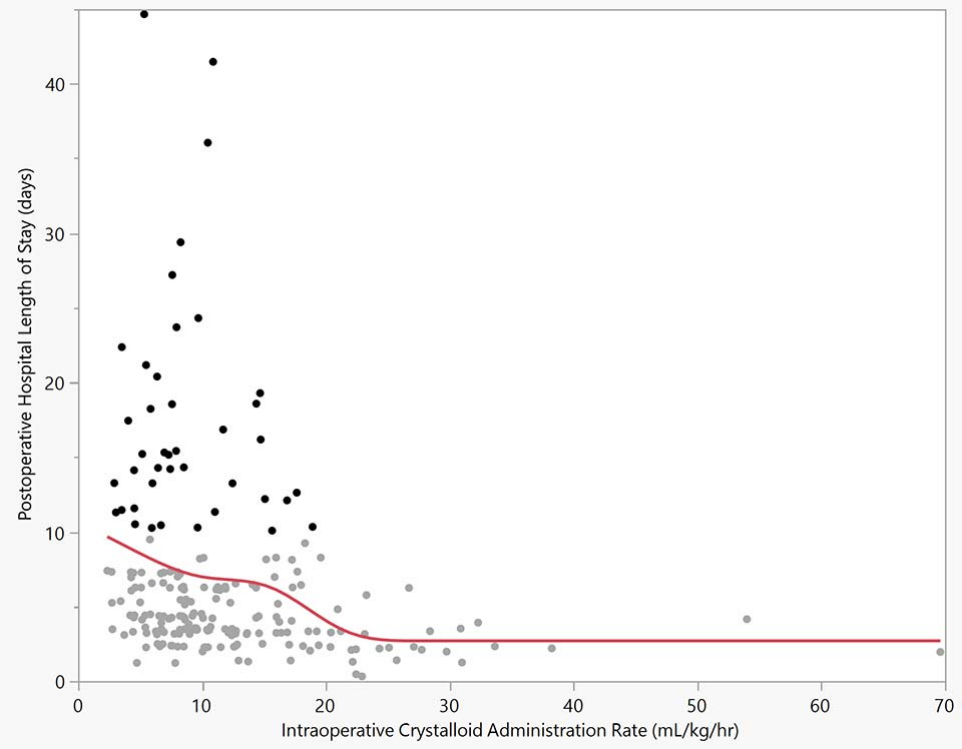
Knotted spline graph showing the postoperative hospital length of stay outliers relative to the intraoperative crystalloid administration rate (mL/kg/hr).

We can also examine the range of postoperative hospital length of stay with another statistical tool, the outlier plot (Figure 2 in this editorial). According to this histogram/outlier plot, the outliers begin to develop when the postoperative hospital length of stay is >10 days. When the outlier values from the histogram/outlier plot are highlighted on the knotted spline graph, all outliers are below the 20 mL/kg/hr intraoperative crystalloid administration rate (Figure 3 in this editorial) with no outliers above 20 mL/kg/hr.

## CONCLUSION

The use of dose-response curves allows investigators to visualize optimal intraoperative crystalloid infusion rates and use these results to develop intraoperative fluid management guidelines for this patient population. This approach provides clinical information that cannot be determined when data are solely presented as summations. This method provides an additional tool to improve our understanding of perioperative medicine.
